# Chyloperitoneum in a toddler on peritoneal dialysis

**DOI:** 10.1007/s00467-024-06597-x

**Published:** 2024-12-02

**Authors:** Veronica Lavelle Bell, Alyssa A. Schlotman, Daniel J. Benedetti, Tracy E. Hunley

**Affiliations:** 1 Monroe Carell Jr. Children’s Hospital at Vanderbilt, Vanderbilt University Medical Center, 2200 Children’s Way, Nashville, TN 37232-9560 USA; 2 Division of Pediatric Hematology Oncology, Vanderbilt University Medical Center, Nashville, TN USA; 3 Division of Pediatric Nephrology, Vanderbilt University Medical Center, Nashville, TN USA

**Keywords:** Chylous ascites, Lymphatics, Hepatoblastoma, Triglycerides, Denys-Drash syndrome, WT1

## Abstract

Chyloperitoneum is an uncommon diagnosis in peritoneal dialysis (PD) patients. While admitted for emesis and feeding intolerance, a 16-month-old male on PD developed milky-colored dialysate with increased triglycerides, indicating chyloperitoneum. In adult PD patients, chyloperitoneum can indicate potentially life-threatening pathologies including malignancies and liver or heart disease. By contrast, pediatric patients on PD with chyloperitoneum had recently undergone PD catheter or gastrostomy tube placement with presumed disruption of abdominal lymphatics. Slowing lymph flow through dietary manipulation and rarely, temporary withholding of PD, resolved chyloperitoneum. We report a toddler on PD with chyloperitoneum in whom abdominal investigation showed multifocal hepatoblastoma. Chemotherapy and a medium chain triglycerides (MCTs)-based diet led to prompt resolution of chyloperitoneum. Intrabdominal malignancy in this patient illustrates the importance of a prompt, thorough evaluation of chyloperitoneum to allow definitive therapy if required.

## Introduction

Cloudy peritoneal dialysate most often indicates peritonitis and training for home peritoneal dialysis (PD) includes coaching to recognize and treat cloudy dialysate. In the absence of increased dialysate leukocytes, other cells and acellular causes of cloudy dialysate must be considered. We describe a child on PD with cloudy dialysate due to elevated triglycerides from chyloperitoneum.

## Case presentation

A 16-month-old male on nightly cycling PD presented to the emergency department for dehydration. He had Denys-Drash syndrome (confirmed *WT1* mutation c. 1300C > T; p.Arg434Cys.) and had undergone bilateral nephrectomy at age 6 months. Other medical problems included anemia, hyperparathyroidism, chronic lung disease, seizure disorder, and gastrostomy tube (G-tube) dependence. He had experienced four days of vomiting with 5–7 episodes of non-bloody, nonbilious emesis per day and was not tolerating G-tube feedings. He had mild cough and congestion, but no fever, diarrhea, or abdominal tenderness. Nightly PD had been continued with low-concentration dialysate which was clear.

He appeared dehydrated with dry lips and mucus membranes. His weight was 7.15 kg down from 7.42 kg 6 days earlier (3.6% weight loss). Capillary refill was normal (< 2 s). His abdomen was non-tender. His PD catheter exit site was in the right upper quadrant and he had a G-tube. Labs showed peripheral leukocytosis to 35,000/µL (absolute neutrophil count 25,760/µL; absolute lymphocyte count 4780/µL), potassium 4.1 mEq/L, phosphorus 8.6 mg/dL, magnesium 3.4 mg/dL, BUN 94 mg/dL, and creatinine 6.73 mg/dL. Hepatic function was normal aside from mildly low albumin (3.2 g/dL). Blood was sent for culture which came back negative. Respiratory PCR evaluation indicated rhinovirus/enterovirus infection, initially thought to explain his symptoms. Intravenous D5 normal saline was started. Feeds were held for 24 h with improvement in vomiting and were slowly advanced based on tolerance, reaching full feeds on day 3. He continued nightly PD.

**Fig. 1:**
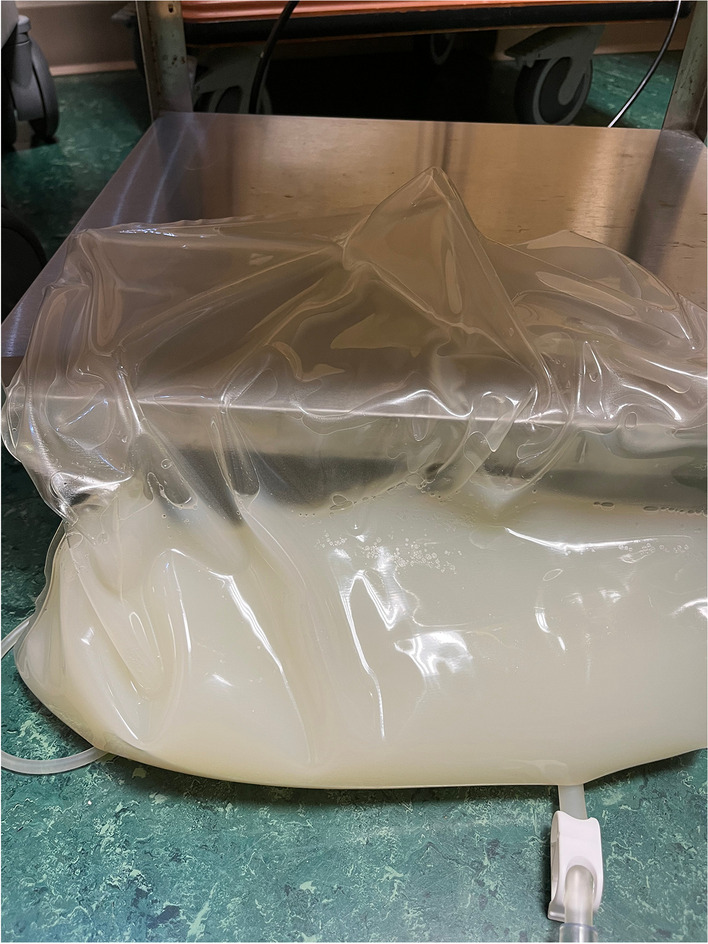
Drained peritoneal dialysate showing a milky appearance due to elevated triglycerides (78 mg/dL). Cell count was normal (40 nucleated cells/µL, 68% neutrophils)

The morning after admission his dialysate appeared faintly cloudy. Dialysate cell count and culture were ordered; results were delayed till the second morning after admission showing 364 nucleated cells/µL, 61% neutrophils. Vancomycin and cefepime were administered intravenous but immediately prior to antibiotics, because the patient remained afebrile, his abdomen non-tender, and his dialysate was clear, dialysate cell count and culture were repeated. This showed 104 nucleated cells/mL, 52% neutrophils. Intraperitoneal (IP) antibiotics were given that night, with repeat dialysate culture obtained upon starting dialysis, and antibiotics were continued the following night. Enteral nystatin antifungal prophylaxis was started. On the third morning after admission, one day into antibiotic treatment, the drained PD fluid appeared milky white (Fig. 1). Dialysate cell count and culture were repeated. Importantly, cell count returned normal (40 nucleated cells/µL, 68% neutrophils). Triglycerides were elevated in the drained dialysate at 78 mg/dL and 122 mg/dL the following day, indicating chyloperitoneum. Blood triglyceride level was normal (130 mg/dL). All four cultures of PD fluid were finalized as negative. Antimicrobials were stopped on the fourth morning of admission, after 48 h of treatment.

Evaluation included an echocardiogram which showed normal flow and velocity at the superior vena cava and normal ventricular function. Abdominal ultrasound revealed multiple liver masses. Computed tomography scan of the chest, abdomen, and pelvis with contrast showed an enlarged liver measuring 13 cm longitudinally at the midclavicular line (normal for age ≤ 10.5 cm) as well as multiple heterogeneously enhancing right-sided hepatic masses, the largest two measuring 6.5 × 6.4 × 7.2 cm and 4.8 × 6.3 × 6.2 cm. The masses were located in liver segments V/VIII. Abdominal or other lymphadenopathy was not detected. Alpha fetoprotein (AFP) was elevated to 37,951 ng/mL (normal 0–41 ng/mL). The disease stage was PRETEXT II, V + , and F + . The core needle biopsy confirmed the diagnosis of hepatoblastoma.

Dietary fat was adjusted from individualized mixed renal formulas to a recipe based on Enfaport (Mead Johnson, Evansville, IN) with lipid content high in medium chain triglycerides (MCTs) (83%). Four days after initiation of neoadjuvant chemotherapy drained PD fluid was visibly clear, and triglycerides were no longer detectable (< 7 mg/dL). His nutrition was changed back to the original recipe at this time without recurrence of chyloperitoneum over the next 3 days of admission or subsequently on PD. He had no further cloudy dialysate or chyloperitoneum while PD was continued until hepatoblastoma resection 3 months later, after which he transitioned to hemodialysis.

## Discussion

PD patients with cloudy peritoneal fluid are appropriately considered to have peritonitis until the diagnosis is confirmed or excluded. In our patient, milky peritoneal fluid developed while undergoing treatment with empiric antibiotics for possible peritonitis. Normal dialysate cell count at the time of milky-colored dialysate prompted consideration of acellular causes of cloudy dialysate and our patient was found to have elevated peritoneal triglycerides indicating chyloperitoneum.

Chyloperitoneum reflects the accumulation of lipid-containing lymph in the peritoneal cavity and is usually discovered by abdominal paracentesis or surgery. In patients on PD, monitoring drained dialysate promptly identifies the color change. Triglyceride levels of 110 mg/dL and 200 mg/dL are cited as thresholds for chyloperitoneum, but PD patients have been diagnosed with lower levels likely reflecting earlier detection and dilution with dialysate. Chyloperitoneum is caused by disturbances in the lymphatic system including malignancy, cirrhosis, trauma, surgical disruption as well as mycobacterial infections, and right heart failure [1]. Because the lymphatic system is a low-pressure network, it is susceptible to compression by tumors, obstruction by infected/infiltrated lymph nodes, or congestion from increased venous pressure of the right heart leading to chyle leak. Developmental abnormalities of lymphatics such as intestinal lymphangiectasia can also cause chyloperitoneum.

Lipids enter the lymphatic system through a single lymph capillary in each small bowel villus (lacteal) which drain into progressively larger mesenteric lymphatic vessels. The intestinal lymphatic trunk, the two lumbar lymphatic trunks from the lower extremities and the lymphatics from the liver coalesce at the cisterna chyli in the abdomen, and the cisterna chyli gives rise to the thoracic duct which transmits lymph back into systemic circulation. The liver is the largest lymph-producing organ accounting for 25–50% of lymph in the thoracic duct [2]. In addition, thoracic duct flow varies with changes in diet: from 1 mL/kg/h during fasting to 200 mL/kg/h with a fatty meal [3]. Notably, in our patient the observation of chyloperitoneum coincided with attaining full feeds on the third morning of admission, allowing sufficient fat intake to indicate and/or exacerbate the chyle leak. Lower fat intake, especially as MCTs, slows lymph flow because MCTs are absorbed into the portal venous system rather than through the lacteals/lymphatics. Decreased lymph flow then allows improved healing of lymphatic disruptions. Importantly, prolonged or recurrent chyloperitoneum is a risk for malnutrition and recurrent infection given ongoing loss of fat and immune cells present in lymph [4].

Adults on PD with chyloperitoneum have similar pathologies as non-PD patients: lymphoma, abdominal malignancies, cirrhosis, pancreatitis, heart failure, and circulatory impairment. Lymphatic disruption may follow the placement of the PD catheter itself, but this affected fewer than 25% of patients in one series [5]. Chyloperitoneum has been seen twice in PD patients with mycobacterial peritonitis: both with late-stage, fatal infection in whom fever and/or fibroadhesive peritoneal inflammation developed, neither of which was seen in our patient or consistent with his course [6, 7]. Chyloperitoneum has also developed in adults on PD after starting calcium channel blockers, particularly lercanidipine and manidipine whose lipophilicity directs them into intestinal lymphatics where they decrease the smooth muscle contractility leading to increased lymphatic hydrostatic pressure [8]. Our patient was not on a calcium channel blocker.

In children on PD, chyloperitoneum has followed traumatic disruption of lymphatics with abdominal procedures including PD catheter placement, gastrostomy, or minor external trauma. MCT-based diets to slow lymphatic flow were used to manage all these patients (see Supplementary references). Inhibition of lipid absorption by octreotide has also been used [9]. Given the potential for ongoing dialysate flow in the abdomen to delay the restoration of lymphatic integrity, cessation of PD was used in some cases with return to PD possible when still required. Our patient had not had recent abdominal trauma or surgery. Echocardiography did not reveal cardiac dysfunction impeding lymphatic return. Abdominal imaging, however, revealed hepatic masses, possibly causing lymphatic disruption through compression of liver or abdominal lymphatics which improved promptly with the reduction in tumor size after chemotherapy.

Chyloperitoneum is seen in a small percentage of patients with cirrhosis and has been reported rarely with liver cancer. Thus, an elderly man with asymptomatic liver disease due to hepatitis B and C diagnosed three years prior, developed abdominal distension over a 15-day period. Paracentesis revealed chyloperitoneum with increased ascitic fluid triglycerides (229 mg/dL). Abdominal CT revealed multiple hepatic nodules, the largest (7 × 7.5 cm) in liver segment V, without intrabdominal lymphadenopathy. Fine needle aspiration revealed hepatocarcinoma [10]. It is noteworthy that our patient’s large hepatoblastoma was similarly located in liver segment V and its rostral complement segment VIII, the most medial segments of the right lobe of the liver. Our patient’s largest mass at maximum dimension 7.2 cm would be much larger relative to his smaller body size compared to this adult hepatocarcinoma patient. The medial location of the masses in our patient and the hepatocarcinoma patient may have been close enough to obstruct liver lymphatics or the abdominal lymphatic trunk as it approaches the cisterna chyli from below. The precise definition of the site of chyle leak in our patient was not pursued with lymphangiography so that tumor treatment could begin as quickly as possible.

Our patient had an elevated peripheral white blood count at admission which we are not able to explain definitively. He was not febrile at any point. While his respiratory pathogen panel by PCR indicated rhinovirus/enterovirus at admission, it is possible that this was not the cause of the full degree of leukocytosis. Blood culture and multiple peritoneal fluid cultures remained negative. C-reactive protein (CRP) and procalcitonin (PCT) are useful serum inflammatory biomarkers which can aid the diagnosis of serious infection. Elevated baseline concentrations in dialysis patients mean that unique threshold levels must be utilized to predict infection in this population [11]. Given the lack of fever in our patient, CRP and PCT were not obtained. An additional emerging index for interpreting infection and inflammation status is the neutrophil-to-lymphocyte ratio (NLR) which can be ascertained from the complete blood count. Like other biomarkers, NLR has recently been combined with CRP to heighten the specificity and sensitivity for the identification of infection in dialysis patients [12]. The NLR on admission for our patient was 5.4 which is within the 90% confidence interval for normal range in 1–2-year-olds [13]. Lastly, it is worth pointing out that earlier reports as well as contemporary experience continue to observe leukocytosis at the time of presentation of some cases of hepatoblastoma [14–18]. It is possible that hepatoblastoma may have contributed to our patient’s leukocytosis.

Despite the predominance of prior treatment success for chyloperitoneum in children on PD with dietary changes and time for lymphatic healing, our case illustrates that serious, even malignant, pathology may be present and that abdominal imaging is warranted to allow definitive treatment.

## Summary

### What is new?

- Chyloperitoneum previously described in pediatric PD patients has resulted from lymphatic disruption with surgery or mild trauma and resolved with dietary changes and time. Our case illustrates that serious, even malignant, pathology may underlie chyloperitoneum, and abdominal imaging is warranted.
